# Complete Genome Sequence of Salinisphaera sp. Strain LB1, a Moderately Halo-Acidophilic Bacterium Isolated from Lake Brown, Western Australia

**DOI:** 10.1128/MRA.01047-18

**Published:** 2018-10-04

**Authors:** Kaela B. O’Dell, E. Anne Hatmaker, Adam M. Guss, Melanie R. Mormile

**Affiliations:** a Biosciences Division, Oak Ridge National Laboratory, Oak Ridge, Tennessee, USA; b Department of Biological Sciences, Missouri University of Science and Technology, Rolla, Missouri, USA; University of Arizona

## Abstract

Salinisphaera sp. strain LB1 was isolated from Lake Brown, Western Australia, surface water enriched at pH 4.0 and with 5% (wt/vol) NaCl. The complete genome sequence is presented in this report.

## Abstract (precis)

Salinisphaera sp. strain LB1 was isolated from Lake Brown, Western Australia, surface water enriched at pH 4.0 and with 5% (wt/vol) NaCl.

## ANNOUNCEMENT

Halo-acidophilic bacteria are gaining attention as a source of enzymes for use in industrial processes that require efficient enzymatic activity within operating conditions at low pH and high concentrations of salt (1). Members of the family Salinisphaeraceae are Gram-negative Gammaproteobacteria that are cocci or short rod shaped and halotolerant (2). Salinisphaera sp. strain LB1 was isolated from surface water from Lake Brown, Western Australia, through enrichment cultures in modified growth medium (3, 4) at pH 4.0 and with 5% (wt/vol) NaCl and plated onto phytagel-solidified medium incubated at 37°C. A pure colony was subcultured in liquid medium for genome sequencing.

Genomic DNA was isolated from Salinisphaera sp. strain LB1 using the Qiagen Genomic-tip 100/G extraction kit and bacterial protocol (Qiagen, Valencia, CA, USA). The genome was generated by the DOE Joint Genome Institute (JGI) using the PacBio sequencing technology (Menlo Park, CA, USA). A PacBio SMRTbell library of >10 kb was constructed and sequenced on the PacBio RS II platform (5), which generated 92,798 ﬁltered subreads for a total of 597,839,667 bases. The reads were then assembled using Hierarchical Genome Assembly Process 3 (HGAP3; v2.3.0.p5) (6). The ﬁnal assembly had an input read coverage of 92.8×. The genome includes a single, contiguous, circular chromosome 4,141,708 bp long, with an average G+C content of 64%. The genome was annotated using Rapid Annotations using Subsystem Technology (RAST) v2.0 (7) and is predicted to include 3,784 protein-coding sequences, 48 tRNAs, and 6 rRNAs in 2 operons. DNA modification detection and motif analysis were performed by JGI using the PacBio single-molecule real-time (SMRT) analysis platform (pbsmrtpipe.pipelines.ds modification motif analysis 0.1.0). Briefly, raw reads were filtered using SFilter to remove short reads and reads derived from sequencing adapters. Filtered reads were aligned to the reference genome for Salinisphaera sp. strain LB1 using BLASR (v5.3) (8). Modified sites were then identified through kinetic analysis of the aligned DNA sequence data (9) and grouped into motifs using MotifFinder. These motifs represent the recognition sequences of methyltransferase genes active in the genome (10). One methylated motif was identified, gAgnnnnnnnnTgcc, which showed 99.2% modification from a count of 830 sites in the genome. All software used the default settings.

Based on the 16S rRNA gene, Salinisphaera hydrothermalis was 96.13% similar over the entire 1,542-bp gene to Salinisphaera sp. strain LB1 when matched using the EzBioCloud 16S database (11). According to JSpeciesWS (12), the ANIb value (calculated by BLAST) of Salinisphaera sp. strain LB1 compared with that of Salinisphaera hydrothermalis C41B8 is 81.15%, and the ANIm (calculated by MUMmer) value is 85.42%, suggesting that strain LB1 and S. hydrothermalis are different species. The complete genome sequence of Salinisphaera sp. strain LB1 will be a critical tool enabling experiments and analyses to uncover mechanisms of adaptation to polyextreme conditions and potential novel enzymes from these environments.

### Data availability.

This complete genome sequence has been deposited at DDBJ/EMBL/GenBank under the accession no. CP029488. PacBio reads were deposited at the NCBI Sequence Read Archive (SRA) under accession no. SRP156246.
